# From light sensing to adaptive learning: hafnium diselenide reconfigurable memcapacitive devices in neuromorphic computing

**DOI:** 10.1038/s41377-024-01698-6

**Published:** 2025-01-03

**Authors:** Bashayr Alqahtani, Hanrui Li, Abdul Momin Syed, Nazek El-Atab

**Affiliations:** 1 Electrical and Computer Engineering Program, Computer Electrical Mathematical Science and Engineering Division, King Abdullah University of Science and Technology (KAUST), Thuwal, Saudi Arabia; 2 Electrical Engineering Department, College of Engineering, Princess Nourah Bint Abdulrahman University (PNU), Riyadh, Saudi Arabia

**Keywords:** Supercontinuum generation, Optical data storage

## Abstract

Advancements in neuromorphic computing have given an impetus to the development of systems with adaptive behavior, dynamic responses, and energy efficiency characteristics. Although charge-based or emerging memory technologies such as memristors have been developed to emulate synaptic plasticity, replicating the key functionality of neurons—integrating diverse presynaptic inputs to fire electrical impulses—has remained challenging. In this study, we developed reconfigurable metal-oxide-semiconductor capacitors (MOSCaps) based on hafnium diselenide (HfSe_2_). The proposed devices exhibit (1) optoelectronic synaptic features and perform separate stimulus-associated learning, indicating considerable adaptive neuron emulation, (2) dual light-enabled charge-trapping and memcapacitive behavior within the same MOSCap device, whose threshold voltage and capacitance vary based on the light intensity across the visible spectrum, (3) memcapacitor volatility tuning based on the biasing conditions, enabling the transition from volatile light sensing to non-volatile optical data retention. The reconfigurability and multifunctionality of MOSCap were used to integrate the device into a leaky integrate-and-fire neuron model within a spiking neural network to dynamically adjust firing patterns based on light stimuli and detect exoplanets through variations in light intensity.

## Abstract (web summary)

Reconfigurable MOS memory enables light-controlled synaptic functions. Integrated into LIF neurons, the device demonstrates potential for dynamic firing patterns in response to light stimuli, promising applications in exoplanet detection.

## Graphical abstract

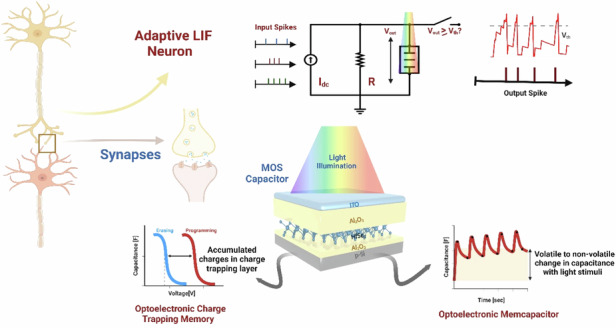

## Introduction

**Fig. 1:**
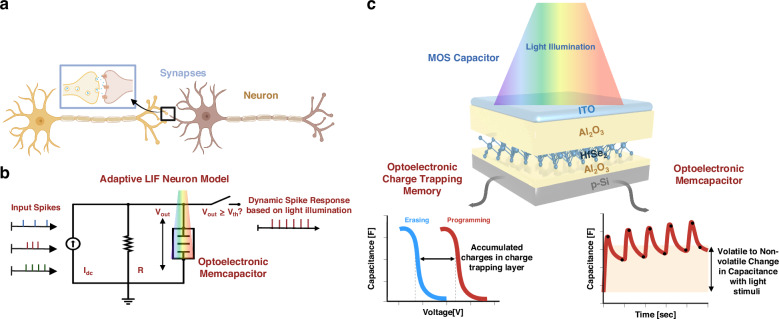
**Neuromorphic systems based on the structure of biological neurons**. **a** Biological neurons interweave via synapses through synaptic chemical messengers, which are transmitted between presynaptic and post-synaptic neurons. **b** The charge-trapping MOS capacitor consists of multiple layers stacked on top of a silicon substrate. Under illumination, this device exhibits non-volatile charge trapping as well as a change in capacitance (i.e., memcapacitor). **c** The adaptive LIF neuron model consists of the input spikes represented as a current source, a leaky resistor, and the optoelectronic memcapacitor device. The light-modulated memcapacitor affects the neuron’s adaptability and the generated output spikes

Replicating synaptic and neuronal structures within optoelectronic devices is crucial for simulating biological functionalities in neuromorphic approaches. This replication allows for advanced behavioral analysis and can facilitate the development of fast and efficient high-density data sensing, processing, and storage, towards “More than Moore”. In 1990, Carver Mead proposed a neuromorphic system that could simultaneously perform computing and memory tasks in a manner similar to that of the human brain^1^. Unlike conventional computers that process data sequentially, the human brain can simultaneously handle multiple tasks, including learning, computing, memory, and perception. The human brain consists of ~100 billion neurons, with each neuron forming connections called synapses^2^. Neurons receive, integrate, conduct, and send electrical impulses to promote the flow of information. Biological neurons interweave via synapses through synaptic chemical messengers, which are transmitted between presynaptic and post-synaptic neurons (Fig. 1a). Emulating the plastic properties of biological neurons can allow these devices to facilitate the development of intelligent and energy-efficient computing systems for applications in various domains. The plasticity of a synaptic connection is its ability to adjust its strength (synaptic weight). Artificial neuron implementation involves using materials with tunable electrical properties or adapting them suitably to external stimuli. By modulating the conductance or capacitance of these artificial synapses, semiconductor neurons can strengthen or weaken their connections based on incoming signals that mimic the synaptic weight.

In conductance synapses, computational processes are used within the current domain, whereas capacitive synapses perform computations within the charge domain^3–8^. Unlike conductance synapses, capacitive synapses exhibit several desirable qualities, such as minimal static power consumption, potential for 3D stacking, and reduced sneak-path current leakage. Most capacitive synapses are implemented by ferroelectric devices, which can be used as memcapacitors or as the gate stack of transistors in non-volatile memory^3^. A memcapacitor exhibits a bias-history-dependent capacitance with electrical biasing^9–11^, or optically as an optoelectronic synapse^12^. Under conditions such as light stimuli, the capacitance of these devices undergoes non-volatile changes to allow the device to be programmed/reset. Despite considerable progress in this field^13,14^, several challenges remain. Because achieving the levels of complexity and efficiency observed in biological systems remains challenging, real-time adaptive neurons have attracted considerable attention. Reconfigurable neurons, whether through electrical biasing or in response to light stimuli, can replicate the multifunctionality found in neuromorphic systems.

Two-dimensional (2D) materials integrated into charge-trapping memories based on mature silicon technology exhibit considerable potential for use in implementing neurons and synapses. Although studies have extensively investigated charge-trapping memories, limited studies have focused on their use as capacitive synapses. Hafnium diselenide (HfSe_2_) has gained considerable attention because of its unique properties such as a 0.9 eV bandgap for a multilayer and a 1.2 eV for a monolayer ~0.3 nm thickness^15^. Studies have demonstrated the capability of HfSe_2_ nanosheets as active layers in memristors^16–18^. The use of 2D nanosheets as a charge-trapping layer (CTL) in two-terminal metal-oxide-semiconductor capacitor (MOSCap) technology is a promising technique for investigating the light–matter interactions of 2D material-based devices. Despite its potential, MOSCap-based memory is rarely used in neuromorphic computing^19^.

In this study, we used a seamless fabrication technique to examine the non-volatile memory capabilities of solution-processable 2D flakes used as a CTL. First, an extensive electrical examination was conducted on these devices to reveal robust and reliable non-volatile memory features. Next, optical characterization revealed the light-sensing capabilities of such devices based on charge trapping and the consequent threshold voltage shift as well as a change in capacitance when exposed to the stimulus, which enabled a multifunctional dual charge-trapping/memcapacitive behavior (Fig. 1b). This memcapacitor mechanism ensured volatility modulation, which enabled the transition from pure volatile light sensing (with no hysteresis) to non-volatile optical data retention. These results were used to develop adaptive neurons that can mimic learning behavior. Thus, the device exhibited light-modulated synaptic function (potentiation/depression) because of charge-trapping and memcapacitive mechanisms. When integrated into the leaky integrate-and-fire (LIF) neuron model (Fig. 1c), the device exhibited adaptability to light stimuli. These dynamic optoelectronic neurons showed exceptional capabilities for detecting exoplanets based on their light intensity in a spiking neural network (SNN). Today’s most advanced SNN models need memory elements that function across various timescales, possessing both short-term (volatile) and long-term (non-volatile) characteristics, ranging from tens of milliseconds to several hours^20^.

## Results

**Fig. 2:**
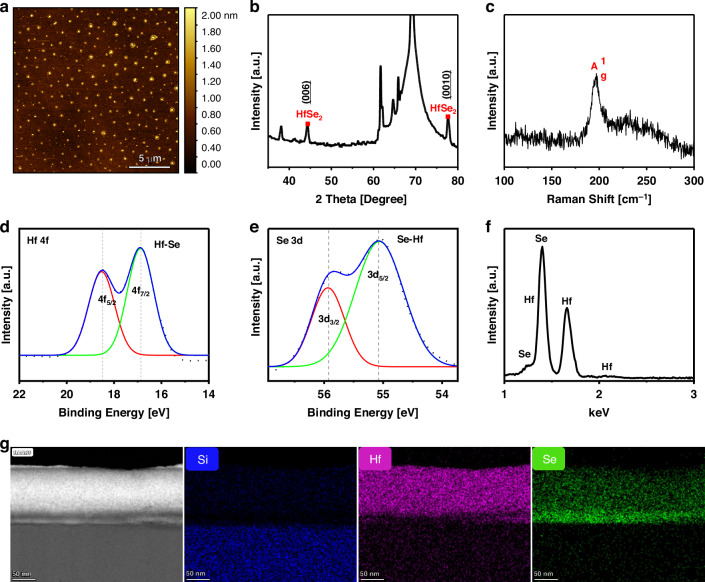
**HfSe**_**2**_ **flake characterization**. **a** Atomic force microscopy scan of the nanosheets. **b** XRD peaks related to the flakes are highlighted in red. **c** Raman spectrum displaying the HfSe_2_ peak at (199 cm^−1^). **d** XPS spectra of Hf 4*f* for HfSe_2_. **e** XPS spectra of Se 3*d* for HfSe_2_. **f** EDX results confirming the material composition. **g** HRTEM of the cross-sectional images confirming the flakes’ material. Scale bar: 50 nm

The MOSCap structure consists of two oxides that enclose the 2D material flakes as CTL (Fig. 1b). The device was fabricated using a layer-by-layer stacking method (see section “Methods” and Supplementary Information 1). Highly doped silicon material was used as the back contact and channel-forming material. High-dielectric material Al_2_O_3_ was used for the tunneling oxide and blocking oxide layers. The HfSe_2_ flakes in isopropyl alcohol (IPA) were sonicated before spin coating to prevent aggregation of the flakes. Atomic force microscopy (a significant indicator) helped optimize the spin-coating technique, flake thickness, and flake distribution uniformity (Fig. 2a). Furthermore, an in-depth structural characterization was conducted to verify the material composition using X-ray diffraction (XRD) and Raman spectroscopy. The peaks presented in red (Fig. 2b) correspond to HfSe_2_ particles, which is consistent with the International Centre for Diffraction Data (ICDD 01-084-6304). The XRD spectra detected the orientation of the (006) and (0010) planes. The Raman spectrum (Fig. 2c) reveals a prominent peak appearing at (199 cm^−1^) corresponding to the ${{\rm{A}}}_{{\rm{g}}}^{1}$ mode, which is related to the 1T phase and is consistent with the finding reported in ref. ^15^. The elemental composition (Fig. 2d–f) was also confirmed by X-ray photoelectron spectroscopy (XPS) and energy-dispersive X-ray spectroscopy (EDX). In Fig. 2d, the binding energy peaks for Hf 4*f* core levels (4*f*_7/2_ and 4*f*_5/2_) are at 16.8 and 18.4 eV, respectively^21,22^. Also, doublet was detected for Se 3*d* **(**Fig. 2e) of 3*d*_5/2_ and 3*d*_3/2_ levels at 55 and 55.8 eV, respectively^15^. High-resolution transmission electron microscopy (HRTEM) was used to obtain cross-sectional images of the flakes (Fig. 2g).

**Fig. 3:**
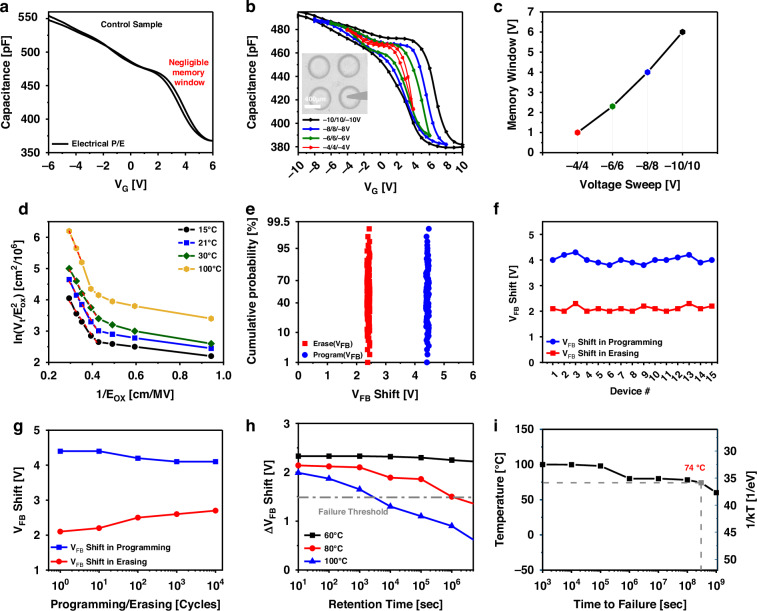
**Device’s electrical performance**. **a** CV measurement of the control sample (without CTL) showing negligible memory window at ±6 V. **b** CV measurement of the device based on 2D material as CTL represents the shift in *V*_FB_ of the P/E cycle at different biasing from ±4 to ±10 V. (inset: optical microscope image of the device under electrical propping). **c** Increment in the memory window with the increase in the basing voltage. **d** Charge tunneling analysis at different temperatures. The primary mechanism responsible for electron emission across the tunnel oxide is F–N tunneling, as indicated by the linear trend at high electric fields. **e** Cycle-to-cycle variability of MOS memory with a standard deviation of 0.028/0.031 of *V*_FB_ shift for P/E cycles, receptively. **f** Device-to-device variability with a standard deviation of 0.14/0.10 of *V*_FB_ shift for P/E cycles on different devices, receptively. **g** Endurance test revealed the memory window reliability after 10^4^ P/E cycles. **h** Temperature-accelerated retention test reveals the device’s performance at 60 °C, 80 °C, and 100 °C temperatures. The device exhibits excellent memory stability when subjected to stressing temperatures of 60 °C and 80 °C. **i** Arrhenius (1/kT) plot extrapolation allows the estimation of the temperature at which the device can operate with memory stability above the threshold for 10 years

The electrical characterization of the MOS memory was conducted by measuring the capacitance–voltage (CV) hysteresis curves. Programming and erasing sweep voltages (P/E) were within the range of ±4 to ±10 V. Therefore, to investigate the change in capacitance in various regions of MOS memory, a sinusoidal signal was applied with an amplitude of 50 mV and a frequency of 100 KHz. The programming of the device involves voltage sweeping from a negative value (−*V*_G_) to a positive value (+*V*_G_) in a forward sweep. Subsequently, the device is erased by sweeping the voltage from the positive value (+*V*_G_) back to the negative value (−*V*_G_) in a backward sweep. The memory window is represented by the difference between the flat-band voltage (*V*_FB_) of the forward and backward sweeps (Δ*V*_FB_)^23^. Based on the principles of the MOS capacitor CV measurements, a flat-band voltage is associated with a change in the capacitance value between the accumulation and depletion states. The control sample (Fig. 3a), which contained oxide only, was also tested to verify that the observed changes in *V*_FB_ were confined to the presence of the 2D material as a CTL. Notably, the measurements (Fig. 3b) revealed a considerable memory window under a low-voltage bias compared with the negligible memory window of the control sample. The increment in Δ*V*_FB_ with the increase in the biasing voltages (Fig. 3c) revealed the effectiveness of charge storage inside these devices with few to sub-nanometer-thickness flakes. The device can reach up to a 6 V memory window at ±10 V biasing. Furthermore, the charge trap states density of accumulated charges was 4.73 × 10^12^ cm^−2^ at ±10 V with an accumulation capacitance of 0.983 nFcm^−2^ and Δ*V*_FB_ = 6 V, with the assumption that the trapped charges in the 2D materials layer/interfaces primarily induced the resulting Δ*V*_FB_ (Supplementary Information 2). The charge emission mechanism was determined by calculating the electric field across the tunneling oxide. The linear trend at high electric fields revealed that Fowler–Nordheim tunneling (F–N) was the primary technique for electron emission in the tunnel oxide under the influence of an electric field. In F–N, electrons traverse a triangular energy barrier, entering the tunnel oxide conduction band. The charges are then swept by the electric field into the CTL. This leads to a corresponding change in the *V*_FB_ of the device. Moreover, the observed linear trend at different temperatures^24,25^, confirms that the F–N tunneling mechanism is still dominant (Fig. 3d and Supplementary Information 3). The cycle-to-cycle and device-to-device variability considering the *V*_FB_ of the P/E cycle are shown in Fig. 3e and 3f, respectively. The MOS memory exhibited excellent endurance of the repetitive rewrites of information and could withstand up to 10^4^ P/E stress cycles (Fig. 3g). This test revealed only a 39% loss in the memory window after P/E cycles. The retention test for non-volatile memories requires considerable time to complete. A common method to shorten the test duration is to accelerate memory retention loss by applying high temperatures and identifying the point at which the device can still retain data above a specific threshold. Thus, a temperature-accelerated test was conducted to determine the retention failure times under stress conditions by examining the memory states after programming at various time intervals and recording the changes in *V*_FB_. Figure 3h displays the retention time of the memory at various temperatures; the failure criterion was set to be less than 1.5 V Δ*V*_FB_. Based on the recorded data, the device exhibited a memory window that remained above the failure threshold for 10^6^ s under stressing temperatures of 60 °C and 80 °C. The recorded retention failure times at various temperatures were plotted as Arrhenius plot (1/kT). Extrapolating the recorded temperature readings (Fig. 3i) reveals that the device can operate at a temperature of up to 74 °C for 10 years (Supplementary Information 4).

**Fig. 4:**
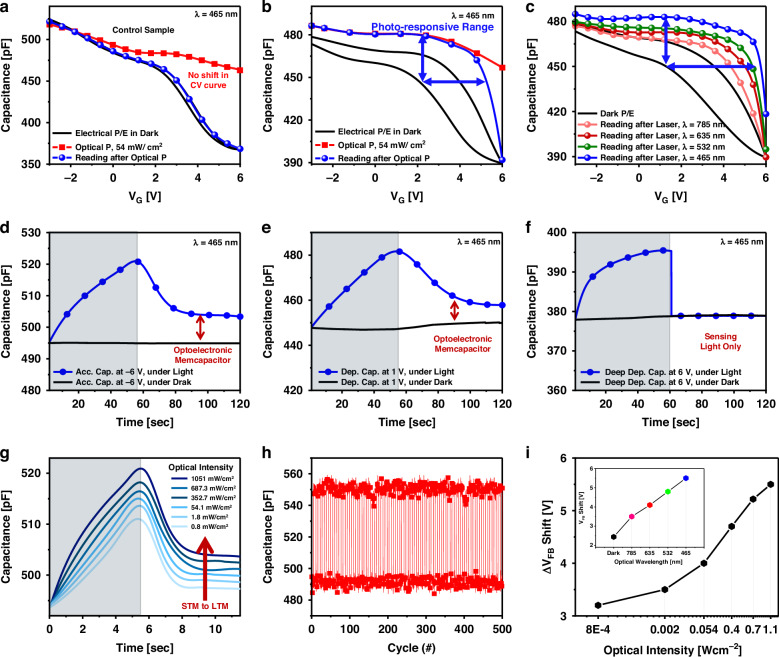
**Optical characterization of the device**. **a** The control sample showed no change after illumination. **b** The MOS capacitor based on 2D material CTL exhibited non-volatile optical data storage represented by the horizontal shift in the *V*_FB_ of the reading curve after optical programming. The upward vertical CV curve shift indicates the memcapacitance mechanism. **c** Different *V*_FB_ shifts were observed in response to various incident light wavelengths. The devices exhibited an 80% window enhancement through blue laser programming at the wavelength of 465 nm from 2 to 3.6 V at ±6 V bias. **d** CT measurement reveals the memcapacitance with the illumination in the accumulation region at −6 V biasing confirming optical data sensing and retention. **e** CT measurement showing a non-volatile change in capacitance with the illumination in the depletion region at 1 V biasing. **f** Deep depletion CT measurement exhibiting volatile light sensing at 6 V biasing. **g** MOS memory CT measurement clarifies the transition from STM to LTM based on optical programming intensity. **h** Memcapacitance device endurance under 500 cycles of optical programming (*λ* = 465 nm/12 ${{\rm{mWcm}}}^{-2}$) in the accumulation region. **i** Memory window enhancement with optical programming intensity (inset: memory window based on the illumination wavelength)

Low programming voltages that preserve light information are highly desirable in optoelectronic memories for achieving secure storage and reducing power consumption. In the operating mechanism of 2D material-based optoelectronic memory, charge-trapping sites are provided for photogenerated charge carriers, which can store carriers even after the removal of light stimuli^26–31^. Thus, the investigation of HfSe_2_ MOS memory under illumination reveals promising results. Optical testing was performed by illuminating the device under the application of a sweep-biasing signal to observe the CV curve under light (i.e., optical programming cycle). Subsequently, the same or a smaller electrical bias was applied to read the stored data in the dark. Optical programming of the control device (without CTL) revealed an unaffected CV curve (Fig. 4a), indicating that the memory state was not altered. By contrast, memory based on 2D materials exhibited non-volatile data retention even after irradiation removal because of charge trapping (Fig. 4b). The use of the HfSe_2_ CTL enhanced the memory window represented by the horizontal curve shift (Δ*V*_FB_) in addition to the detection and storage of optical data. The band diagram was used to explain the mechanism of the increased amount of stored charge in the trapping layer (Supplementary Information 5). Specifically, the photons from light stimulus create electron–hole pairs in the CTL. These pairs become trapped at the interface between the charge-trapping and dielectric layers^32^. The trapped electrons and holes increase the amount of charge stored in the trapping layer, which results in data retention. Furthermore, the devices were examined under various laser wavelengths in the visible range (Fig. 4c): 465, 532, 635, and 785 nm for the same bias voltage, illumination time, and intensity. Because of the increased number of photoionized electrons, the higher incident photon energies caused a larger shift, which could be attributed to various charge-trapping/de-trapping physics^32^. The device has a non-volatile memory potential with an 80% window enhancement by optical programming from 2 to 3.6 V at ±6 V bias. According to a previous study, photoionized electrons in Al_2_O_3_ layers can be disregarded^33,34^.

The optical testing showed identifiable features that differed from the conventional CV characteristics observed in MOS memory. A flat-band voltage change in MOS memory is often observed due to electrical charging and represented by horizontal shift. The results demonstrated an increase in the accumulation capacitance, followed by a further increase in the depletion capacitance under illumination as the CV curve shifts vertically (Fig. 4b). The change can be retained after light removal based on biasing from the accumulation to depletion regions in the 2D material-based memcapacitor as shown in capacitance–time (CT) measurement (Fig. 4d, e). However, the capacitance of the deep depletion region was only sensitive to light and returned to its original (dark) value when the light was removed (Fig. 4f). The control sample exhibited only light sensing in the deep depletion and no change in capacitance in other regions (Supplementary Information 6). This phenomenon of 2D material MOS memory revealed the volatility modulation of the in-memory optical sensor based on the biasing region. Thus, the build-up of photo-excited excess carriers at the heterointerfaces of the oxides and 2D materials explained the memcapacitance effect of light programming^35,36^. According to the intensity of light exposure, these devices showcased the transition from short-term memory to long-term memory (STM to LTM), as depicted in Fig. 4g. Increasing the light intensity plays a role in increasing the decaying time constant which slows the decaying curve and results in longer data retention (Supplementary Information 7). The endurance of the light-controlled memcapacitor device in the accumulation region exceeded 500 cycles (Fig. 4h). These observations indicate an intriguing memcapacitive mechanism and the charge-trapping mechanism with optical programming. It also demonstrates an improvement in the memory window of such a device (Fig. 4i). Charge trapping can be achieved in 2D material flakes through both electrical and optical methods, whereas the memcapacitive mechanism in this context is facilitated through optical programming only.

## Discussion

**Fig. 5:**
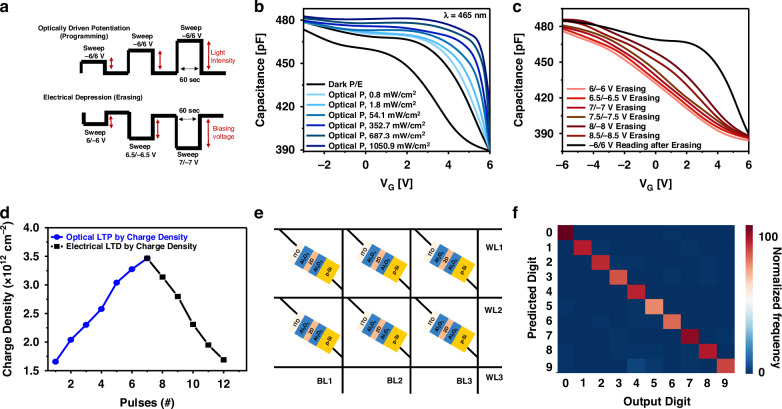
**Capacitive synaptic behavior. a** Optical/electrical pulses are used for potentiation, whereas electrical pulses are used for depression. **b** Device exhibited a range of light-induced LTP in response to distinct illumination intensities. **c** LTD was observed based on electrical erasing signals from 6 to 8.5 V with a step of 0.5 V. **d** Capacitive synaptic weights change based on LTP/LTD. **e** The schematic of the capacitive synaptic array circuit. **f** The accuracy variation of handwritten digit recognition is higher than 96% at 20 epochs

An artificial synapse behaves according to two types of synaptic plasticity, namely long-term potentiation (LTP) and long-term depression (LTD)^37^. The device exhibited optoelectronic synaptic features that were influenced by the light-modulated charge-trapping mechanism. Distinct illumination settings result in MOS memory exhibiting various light-induced LTP. These parameters included electrical triggering, photon energy at varying wavelengths, and illumination intensity. For instance, LTP and LTD were emulated by gradually tuning the capacitance state of the MOS memory device (Fig. 5a). LTP is achieved by changing the incident light intensity because the light intensity determines the total quantity of photogenerated charge carriers^32^ and is frequently related to the possibility of charge trapping or interfacial physics. Therefore, adjusting the light intensity can achieve multiple storage states (Fig. 5b). However, LTD can be performed by erasing the memory electrically, incremental erasing voltage sweeps from 6 to 8.5 V with a step of 0.5 V (Fig. 5c). The device achieved variations in the synaptic weights for potentiation and depression based on capacitance values (Fig. 5d). Therefore, the system-level performance of such devices as an Optoelectronic non-volatile synapse is estimated for hardware realization of neuromorphic architecture (Fig. 5e). SnnTorch is a Python library that makes it easier to implement gradient-driven learning in SNN^38^. It seamlessly integrates per-designed spiking neuron models into the PyTorch environment, where they function as recurrent activation units. To showcase the potential of training a multilayer, fully interconnected SNN with gradient descent for image classification tasks, we utilized the MNIST dataset. The simulations achieved a classification accuracy of over 96% in recognizing handwritten digits (Fig. 5f).

**Fig. 6:**
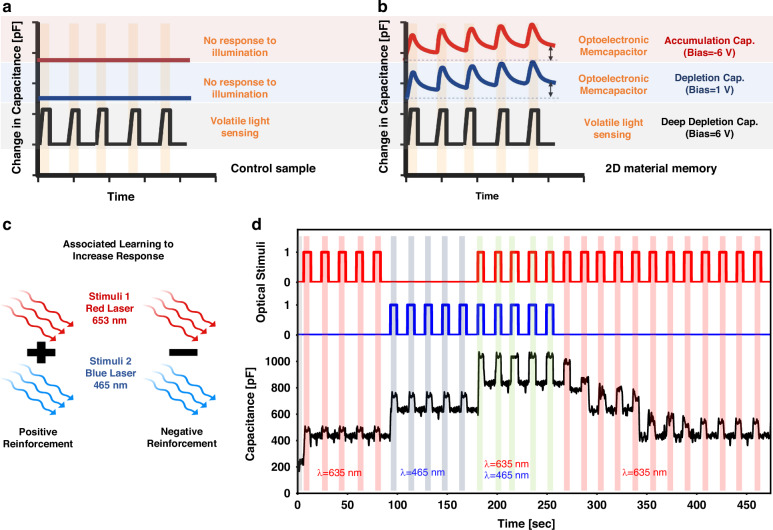
**Associated learning with memcapacitor. a** An illustration of the control sample (without CTL) behavior under illumination, presenting various MOSCap regions. **b** Optoelectronic memcapacitor exhibited modulated light sensing based on the MOSCap region. **c** The learning process involves stimuli association, which strengthens synapses and enhances responses. Negative reinforcement occurs on association removal. **d** When testing a device with a red laser (*λ* = 635 nm) in the accumulation region, its memcapacitive response improved on being influenced by a blue laser (*λ* = 465 nm) due to associated learning. However, when the association was removed, the response of the device to the red laser gradually decreased over time (while illumination and in the dark) until it reached its previous level. The improvement was temporary and dependent on associated learning

The memcapacitor mechanism was further explained through CT measurements using optical programming (Fig. 6a, b). This event-driven memcapacitor was used to illustrate separate stimulus-associated learning. Associated learning (Fig. 6c) can be used to increase the responses of these optoelectronic synapses. This learning process involves stimuli pairing, which results in a strengthened synapse and enhanced response to the stimulus. This response can be enhanced through repeated exposure to light stimuli. Figure 6d represents the memcapacitive response in the accumulation region incorporating associated learning. The device response to the red laser (*λ* = 635 nm) improved because of the influence of the blue laser (*λ* = 465 nm). On removing this association, the response to the red laser gradually decreased over time to its previous level.

**Fig. 7:**
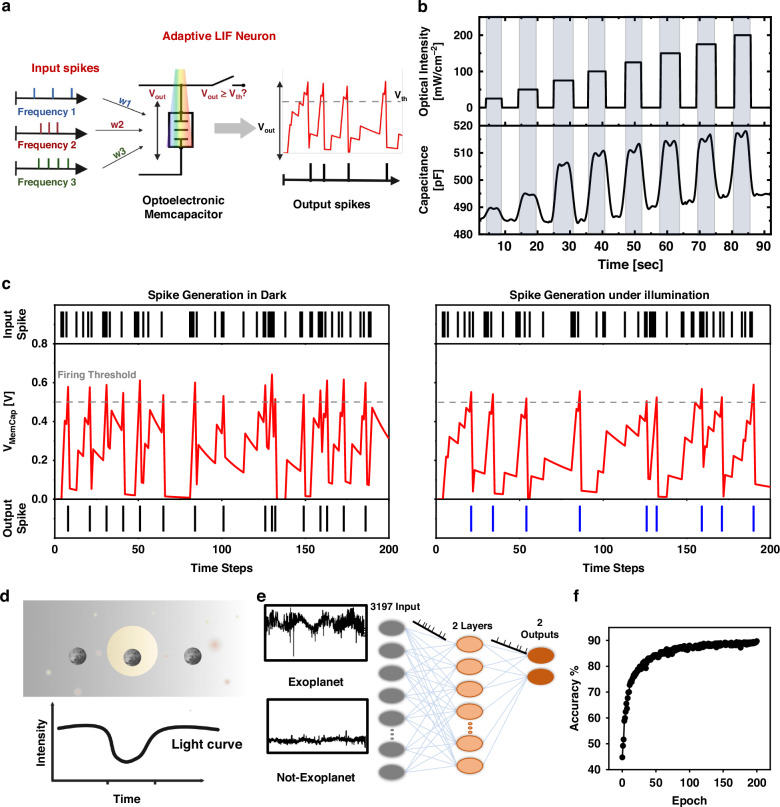
**Adaptive LIF Neuron and exoplanet detection. a** A certain spike frequency is generated by the LIF neuron from the other neurons’ input with varying spike frequencies. The memcapacitor’s electrical and optical programming implies the neuron can dynamically adjust its response qualities. **b** During the CT measurement under varying light intensity (465 nm), the capacitance values of the memcapacitor alter considerably achieving eight levels. **c** The results of the simulation demonstrate that the light-modulated memcapacitor exhibits an adaptive behavior. The increased capacitance values reduce the output spikes when the device is illuminated (465 nm, 54.1 mWcm^−2^). The higher capacitance values cause the charging and discharging time to slow down, resulting in fewer spikes. **d** The transit method exhibits periodic fluctuations in a star’s brightness caused by an orbiting exoplanet crossing in front of it. **e** Dynamic illumination sensing neuron in the SNN for exoplanet detection. Each neuron receives input signals from a target star’s temporal light intensity variations. **f** The testing accuracy over increased epochs reached 90%

Light-modulated memcapacitor potentials can be used to develop adaptable neurons that can replicate learning behaviors. To implement an adaptive neuron, a memcapacitor was integrated into the LIF neuron model (Fig. 1c) instead of a conventional capacitor. The LIF neuron receives inputs from other neurons with different spike frequencies and generates an output spike with a specific frequency based on the accumulation and extraction parts of the neuron. It released a spike if the integrated voltage exceeded a certain threshold. The ability of a memcapacitor to be electrically and optically programmed indicates that the neuron can undergo adaptive changes in its response properties. This phenomenon could lead to learning-like behavior, where the neuron’s spike frequency adapts over time based on the patterns of the light stimuli it receives (Fig. 7a). The potential dynamics in the LIF neurons were calculated using the following mathematical equation^38^:

1 $${{\tau }}\frac{{\rm{d}}{{V}}_{{\rm{out}}}({{t}})}{{\rm{dt}}}=-{{V}}_{{\rm{out}}}\left({{t}}\right)+{{R}}{{{I}}}_{{\rm{dc}}}\left({{t}}\right)$$

2 $${{\tau }}={{R}}{{{C}}}_{{\rm{MemCap}}}$$

where *τ* is the time constant and can be calculated as indicated in Eq. (2), *V*_out_ (*t*) is the voltage across the memcapacitor over time, *R* is the leaky resistance, *I*_dc_(*t*) is the input current spikes over time, and *C*_MemCap_ is the memcapacitor.

The speed of charging or discharging of a capacitor depends on the amount of capacitance that increases or decreases depending on *τ* in Eq. (2). The capacitance values of the memcapacitor changed considerably under varying light intensities achieving eight levels according to the intensity variations with minimum Δ*C*_MemCap_ = 2 pF (Fig. 7b). These values were then used to simulate Lapicque’s RC neuron model^38^. The simulation exhibited adaptability in response to light stimuli in which the frequency of the generated spikes was dynamically altered in accordance with the intensity of light, in contrast to that in dark conditions (Fig. 7c). In neuromorphic computing architectures, adaptive neurons are integrated into larger systems for high-efficiency computational tasks. Adaptive neurons facilitate pattern recognition, associative memory, and autonomous learning. Therefore, using adaptive neurons in SNNs for exoplanet detection using the transit method, which relies on changes in light intensity, is an innovative approach that incorporates neuroscience, astronomy, and semiconductor technologies.

The transit method (Fig. 7d) involves observing periodic dips in the brightness of a star as an orbiting exoplanet passes in front of it, which blocks a fraction of the star’s light^39^. Conventional methods for analyzing transit data typically involve complex algorithms and extensive computational resources. However, by using SNN with adaptive neurons, we can achieve efficient and accurate detection of exoplanets with simultaneous reduction of computational complexity^38^. This approach involves adaptive neuron modeling based on the behavior of biological neurons. These neurons can adjust their sensitivity and response characteristics based on incoming signals, which enables them to adapt to the varying light intensity patterns associated with exoplanetary transits. The SNN architecture for exoplanet detection (Fig. 7e) consisted of layers of adaptive neurons interconnected through synapses (section “Methods”). Each neuron receives input signals corresponding to temporal variations in light intensity observed from a target star. When the light curve data are input into the network, the neurons dynamically modulate their firing rates and synaptic strengths in response to the detected patterns. Adaptive neurons are used in SNN for exoplanet detection because they can operate in real-time and adapt to changes in input conditions, with a high accuracy of ~90% at 200 epochs (Fig. 7f).

Table 1 presents a comparative analysis of previous capacitive memory devices and their distinguishing features (see Supplementary Information 8 for further details). Notably, this study showcased two operating mechanisms, namely MOS memory charge-trapping and light-regulated memcapacitance. This combination exhibits considerable potential for integration into neuromorphic systems, distinguishing the device from existing capacitive devices. Various MOSCap regions and their light responsiveness exhibited a modulated volatility memory device based on the applied voltage.

**Table 1:** Previously reported capacitive memory devices and their neuromorphic applications

Device type	Working mechanism	Capacitive memory modulation	Synaptic functions	Neuron simulation	Memory volatility	Ref.
Ferroelectric capacitor	Memcapacitor	Electrical	Yes	No	Non-volatile	^4,5,41^
Gate-to-source/drain capacitance in FET	Memcapacitor	Electrical	Yes	No	Non-volatile	^6^
Oxide-based capacitor	Memcapacitor	Electrical	Yes	No	Non-volatile	^7^
Metal–semiconductor–metal	Memcapacitor	Electrical and optical	Yes	No	Non-volatile	^12^
MOS capacitor	Charge trapping/*V*_FB_ shift	Electrical and optical	Yes	No	Non-volatile	^19^
This device	Charge trapping/*V*_FB_ shift and memcapacitor	Electrical and optical	Yes	Yes	Volatile to non-volatile	–

To conclude, an optoelectronic MOS charge-trapping memory is an example of the excellent potential of mature Si-based memories. The CV curves revealed a reliable memory window and impressive endurance and retention under both electrical and optical/electrical testing. Moreover, the device exhibited a memcapacitive mechanism that allowed for analog-like sequential changes in capacitance in response to an optical stimulus with eight distinguished levels. The CT curves also demonstrated this trend of capacitance change, providing insight into the capabilities of the device. The responsiveness of the device to light across the entire visible spectrum is notable and its volatility was modulated from non-volatile optical storage to volatile optical sensing based on the biasing region of the MOS capacitor. This phenomenon resulted in the device exhibiting neuromorphic characteristics, including synaptic as well as adaptive neuron features. Finally, using this multifunctionality, the device was used as an adaptive neuron for dynamic real-time light intensity detection within the SNN.

## Methods

### Hafnium diselenide flakes material characterization

A dimension icon scanning probe microscope was used to determine the distribution and thickness of the solution-processable HfSe_2_ particles. Additionally, the flakes were analyzed by Raman spectroscopy using a WITec Apyron instrument with a 532-nm laser. The crystal structures of the flakes were determined using a Bruker D2 phase XRD system. The flake elemental composition was studied by Supra XPS and Helios EDX. A high-resolution transmission electron microscope (FEI Titan Cs Probe HRTEM) was used to observe the cross-sectional structure and the STEM EDS mapping for the material composition of the flake.

### MOS capacitor fabrication

The fabrication process was performed according to cleanroom standards and special precautions were taken to avoid contamination. HfSe_2_ MOS devices were fabricated on Boron-doped, p-type Si (100) wafers with a resistivity of ~0.01–0.02 Ω-cm. The back contact and channel-forming materials were the substrates. The preparation steps included RCA cleaning and immersion in a buffered oxide etchant solution to eliminate impurities and native oxides from the silicon surface. Next, the sample was coated with a 4-nm thick tunneling oxide layer of high-dielectric Al_2_O_3_ using atomic layer deposition at 250 °C. The 2D material flakes were sonicated in IPA for 3 min before being coated onto the substrate using the spin-coating technique. A blocking oxide layer of Al_2_O_3_ (12-nm thickness) was deposited. Finally, indium tin oxide was sputtered as a top contact using a shadow mask to form 400 μm diameter circles.

### Electrical and optical characterization

An Agilent B1500A semiconductor device parameter analyzer was used for the electrical characterization of the devices. The optical investigations used a range of visible light-emitting diode sources with wavelengths of 456, 532, 635, and 785 nm. A commercial UV-enhanced Si photodetector (Newport Corporation) was used to measure and calibrate the illumination power.

### Neuron simulation and implementation in the SNN

Lapicque’s RC model^38^ was used to implement the adaptive neuron and study the behavior of neurons by changing the capacitor values. This LIF neuron model was simulated by the UCSC Neuromorphic Computing Group. The two neuron models were compared based on memcapacitor experimental readings in the dark and under illumination. SnnTorch was used to integrate these neurons. SnnTorch is a Python library for deep learning that allows gradient-based optimization of the SNN. The network contained three linear and three leaky layers that were fully connected and used as the neural network architecture. The SNN parameters include 3197 neurons in the input layers, the middle linear layer contains 64 neurons, and the output layer takes 32 inputs and outputs two results (planet with/without an exoplanet). The output for classification was selected as the output neuron with the highest firing rate. The data forwarded to the network describe the variations in the flux (light intensity) of thousands of stars. Each star was assigned a binary label of either 1 or 2. The data identified as number 2 confirmed the presence of at least one exoplanet orbiting the star. This dataset was preprocessed using the synthetic minority oversampling technique to balance the class distribution, as applied by Lin et al. in ref. ^40^.

## Supplementary information

Supplementary information

## Data Availability

The corresponding author can provide the supporting data upon reasonable request.
