# Supplementary material for: From light sensing to adaptive learning: hafnium diselenide reconfigurable memcapacitive devices in neuromorphic computing
Source: Light Sci Appl. 2025 Jan 3;14:30. doi: 10.1038/s41377-024-01698-6 (PMC11697364; doi:10.1038/s41377-024-01698-6)
Supplement: Supplementary file 1 — Supplementary information [file 41377_2024_1698_MOESM1_ESM.docx]

Supplementary Information for

**From Light Sensing to Adaptive Learning: Hafnium Diselenide Reconfigurable Memcapacitive Devices in Neuromorphic Computing**

*Bashayr Alqahtani^1,2^,* *Hanrui Li^1^, Abdul Momin Syed^1^, and Nazek El-Atab^1^**

^1^ Electrical and Computer Engineering Program, Computer Electrical Mathematical Science and Engineering Division, King Abdullah University of Science and Technology (KAUST), Thuwal, Saudi Arabia. Email: [nazek.elatab@kaust.edu.sa](mailto:nazek.elatab@kaust.edu.sa).

^2^ Electrical Engineering Department, College of Engineering, Princess Nourah Bint Abdulrahman University (PNU), Riyadh, Saudi Arabia.

Supplementary Information 1: The MOS device fabrication process.

Supplementary Information 2: The density of charge trap states.

Supplementary Information 3: The charge emission mechanism identification.

Supplementary Information 4: Temperature-accelerated retention test.

Supplementary Information 5: The band diagram of the MOS memory structure.

Supplementary Information 6: Memcapacitance of Aluminum as the top electrode.

Supplementary Information 7: Decaying time constant measurement for STM to LTM.

Supplementary Information 8: Recent advancements in research on capacitive memories.

Supplementary Information 1: The MOS device fabrication process, is illustrated in Fig. S1. The highly doped silicon substrates with a thickness of 525±25 µm, p-type are used as the back contact and channel-forming material. On Boron-doped, p-type Si (100) wafers with the resistivity of ~0.01-0.02 Ω-cm, deceives of hafnium Diselenide (HfSe_2_) as CTL were fabricated. Prior to loading into the ALD tool for the oxide deposition, samples were prepared by RCA clean and dipped into BOE solution to remove impurities and Si native oxides. The RCA cleaning involves three minutes of sonication in an Acetone bath at 10% ultrasonic power and 45 kHz, flowed by IPA bath and the same sonication process, and DI water cleaning then nitrogen blow drying. After that, a tunneling oxide layer with a 4 nm thickness of Al_2_O_3_ high-dielectric material was produced onto the sample using ALD at 250 °C using trimethylaluminum as precursor and water. An ellipsometer was used to measure the film thickness, which was determined to be 4 nm across all samples. After ALD, the 2D material flakes in IPA were sonicated for 3 min. to avoid the aggregation of flakes. Then, the nanosheets were deposited by a spin-coating process (500 rpm speed and 100 accelerations for a minute). To remove the IPA residuals, samples were annealed on a hot plate at 80 °C for a minute. Later, a 12 nm thickness of Al_2_O_3_ was grown on top of the samples as the blocking oxide layer at 250 °C by ALD. The top contact was then sputtered using a showed mask to form circles of 400µm diameter of Indium Tin Oxide (ITO) and 80 nm thickness. The Al sputtering was at room temperature and 80 DC current.


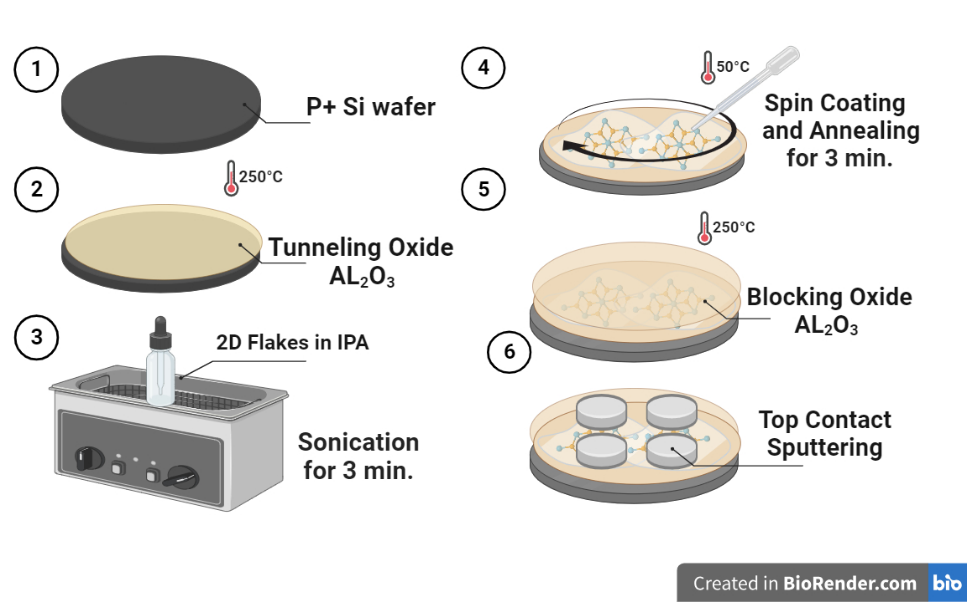


**Fig. S1** **The device fabrication process**.

Supplementary Information 2: The density of charge trap states (N_t_) can be calculated using Equation 1.1, considering that ΔV_FB_ is primarily due to the charges trapped within the charge trapping layer (CTL).

$N_{t}=\frac{C_{t}\times\Delta V_{FB}}{q}$ (1.1)

Where: N_t_ is the density of charge trap states, C_t_ is the accumulation capacitance per unit area, ΔV_FB_ is the shift between the flat-band voltage of both P/E curves and q is the elementary charge. ^1^

**Fig. S2 The charge trap states density at different biasing.**

Supplementary Information 3: By studying and computing the electric field across the TO, it can be attempted to determine the charge emission mechanism, using Equation 1.2 and considering the electric field that passes the three layers TO, BO, and CTL.

$E=\frac{V_{g}}{t_{TO}+t_{BO}\left( \frac{\varepsilon_{TO}}{\varepsilon_{BO}} \right)+t_{CTL}\left( \frac{\varepsilon_{TO}}{\varepsilon_{CTL}} \right)}+\frac{{qN}_{t}}{\varepsilon_{TO}+\varepsilon_{BO}\left( \frac{t_{TO}}{t_{BO}} \right)+\varepsilon_{CTL}\left( \frac{t_{TO}}{t_{CTL}} \right)}$ (1.2)

Where: E is the electric field, V_g_ is the applied biasing voltage, q is the elementary charge, N_t_ is the charge trap density, t is the layer thickness, and ε is the dielectric permittivity. ^2^

In Fowler-Nordheim tunneling (F-N), electrons traverse a triangular energy barrier, entering the TO conduction band. These charges are then swept by the electric field into the CTL. This leads to a corresponding change in the V_FB_ of the device. The emission rate of charges during F-N tunneling is characterized by Equation 1.3:

$J= C_{1}E_{ox}^{2} e^{{C_{2}}/{E_{ox}}}$ (1.3)

Where: J is the F-N tunneling current, E_ox_ is the electric field across the TO, and C_1_ and C_2_ are

constants in terms of the effective mass and barrier height. ^2^

In Fig. 3d, The natural logarithm of the change in V_FB_ divided by the electric field's square is plotted against the reciprocal of the electric field. The observed linear trend after linear fitting indicates that F-N tunneling is the predominant mechanism driving electron emission through the tunnel oxide. ^2–6^ To further validate the F-N tunneling mechanism, the temperature-dependent measurements are conducted by programming/erasing the device at different temperatures 15 °C, 21 °C, 30 °C and 100 °C. ^7,8^

Supplementary Information 4: Temperature-accelerated retention test.

To measure retention time, a commercially acceptable Arrhenius plot method was used. The device was first programmed in an atmospheric environment, and its initial flat band voltage was recorded. The device was then baked at a specific temperature. After cooling to ambient temperature, its CV measurement was taken. The experiment tracked the change in the device's flat band voltage with cumulative baking times at 60, 80, and 100 °C. The retention failure times at these temperatures were plotted using an Arrhenius (1/kT) plot. By extrapolating these values, the temperature at which the device can still perform after 10 years was estimated.

Supplementary Information 5: In order to comprehend the mechanism of charge trapping, the band diagram of the MOS memory structure is presented for HfSe_2_ in Fig. S3. The charge trapping layer (CTL) of HfSe_2_ has an electron affinity of 4 eV. ^9^. The band gap of the ALD-grown aluminum oxide is 6.5 eV, and the electron affinity is 1.6 eV. ^10,11^ The electron affinity and bandgap for a silicon substrate are 4.05 eV and 1.12 eV, respectively. The CTL has a similar bandgap and electron affinity as Si, and the alumina oxide (tunneling layer) contributes to the electron and hole barrier heights of 2.45 eV and 2.93 eV, respectively. Therefore, it is preferable for electrons to tunnel through the oxide layer due to the lower barrier height. During a positive biasing, the barrier for the substrate's minority carriers (electrons) becomes thinner, allowing electrons to cross the oxide and be stored at CTL. In contrast, holes with a negative gate bias are anticipated to tunnel the barrier for the same reason.


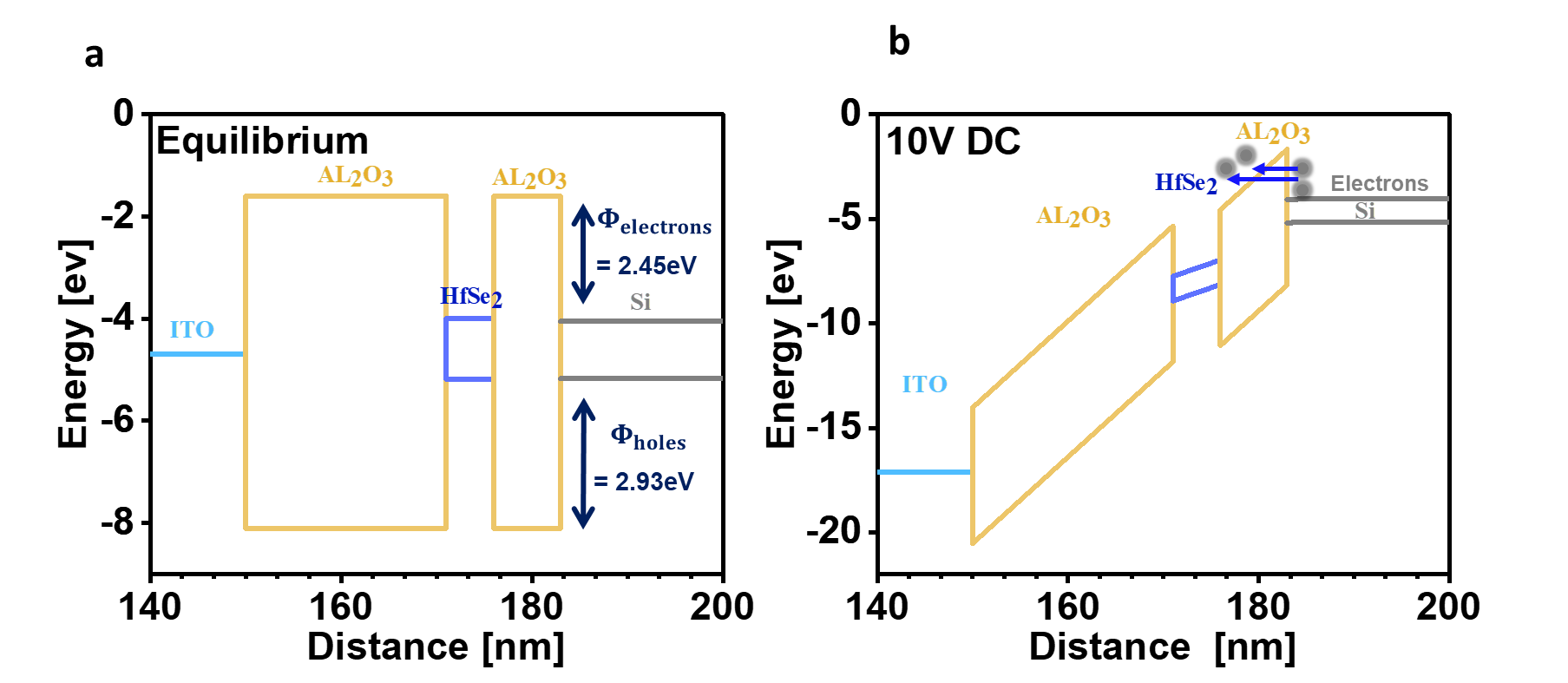


**Fig. S3** **The MOS memory energy band diagram**. a Energy band diagram under no bias. The alumina oxide (tunneling layer) results in electron and hole barrier heights of 2.45 eV and 2.93 eV, respectively, with silicon. b Schematic illustration of F-N tunneling of charge carriers through a triangular barrier during the programming cycle.

Supplementary Information 6: To explore the memcapacitive mechanism discovered in this study, it is suggested to fabricate other devices with Aluminum (AL) as the top electrode. In Fig. S4, the Capacitance-Time (CT) measurement shows that the control devices (Fig. S4a and S4c) with ITO in (a) and AL in (c) both exhibited only optical sensing in the inversion capacitance. The devices shown in Fig. S4b and Fig. S4d have a 2D material nanosheet as the Charge Trapping Layer (CTL), with an ITO top contact and AL, respectively. These devices exhibit a change in capacitance due to the charge tapped in between the interfaces when exposed to light. This change in capacitance can either be retained after light removal or erased by reverse biasing from the accumulation to depletion regions. This implies that the modulation of memcapacitance with light is independent of the top contact material, although ITO provides better light detection and higher response. On the other hand, AL as top electrode showed better electrical conductivity and larger memory window when performing the Capacitance-Voltage (CV) measurement.


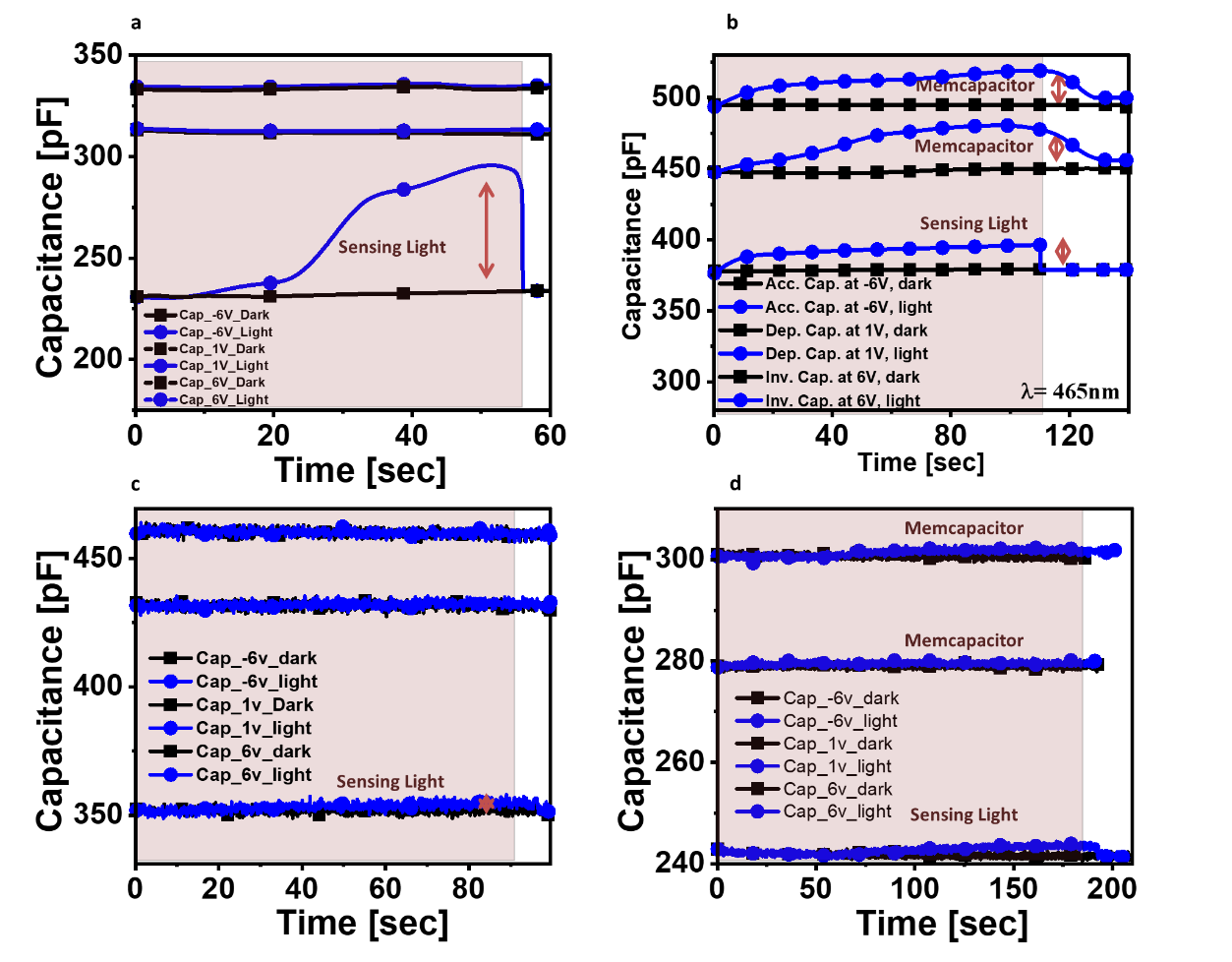


**Fig. S4 The light-modulated memcapacitance of devices. a** The control device with only oxide and no charge trapping layer, and ITO as the top contact, does not exhibit memcapacitance**. b** The device based on 2D material with ITO as top contact exhibits memcapacitance in both accumulation and depletion states. **c** The control device that has AL as the top contact does not show any memcapacitance. **d** The 2D material device with AL top contact displays memcapacitance in both accumulation and depletion states.

Supplementary Information 7: Decaying time constant measurement for STM to LTM.

Today's most advanced Spiking Neural Network (SNN) models require memory elements that can operate across a wide range of timescales. These memory elements must exhibit both short-term (volatile) and long-term (non-volatile) characteristics, allowing them to function effectively over periods ranging from tens of milliseconds to several hours. It is evident that pulse amplitude and duration are significant factors influencing the transition. In biological systems, short-term facilitation occurs on the millisecond scale, while long-term facilitation manifests as an increase in both short-term facilitation and the baseline level of transmitter release. ^12^ The amplitude increases in response to higher stimulation, whether through pulse intensity, duration, or repetition, thus demonstrating the transition. In memristor-based systems^13,14^, these transitions are observed to occur within milliseconds. The transition from STM to LTM was observed in the memcapacitor devices in the accumulation region from less than a second to a few seconds. This transition is critical for adaptive behavior in neuromorphic systems. Specifically, the time constant of the decaying curve, which follows optical programming, provides insight into this transition, when the device was optically programmed at an intensity of 0.8 mWcm^-^², the time constant of the decaying curve was 0.78 seconds. Also, when programmed with a higher intensity of 1.8 mWcm^-^², the time constant increased to 1.2 seconds (Fig. S5). These data, shown in **Table 1,** indicate the duration over which the device retains the programmed state. The ability to adjust the time constant through varying light intensities demonstrates the device's capacity to emulate both short-term and long-term memory functions, which is essential for the dynamic and adaptive nature of SNNs. It should also be noted that not only the time constant but also the capacitance value increases with light intensity. Moreover, the capacitance value does not return to its original state. The increased capacitance value, which persists for tens of seconds, confirms the enhanced retention and the transition from shorter term memory to longer term memory (as shown in **Fig. S5**). Long-term memory functions are also proven through the stable V_FB_ shift of these devices when reading their state after illumination.

**Table 1|** Time constates of the memcapacitor curves under illumination at various light intensities.

| Illumination intensity  [mWcm^-2^] | The time constant after exponential fitting  [sec] |
| --- | --- |
| 687.3 | 1.7 |
| 352.7 | 1.5 |
| 54.1 | 1.3 |
| 1.8 | 1.2 |
| 0.8 | 0.78 |

**Fig. S5 Transition from STM/LTM.** Exponential fitting curves^15^ to find the time constant of CT measurements after various optical programming intensities are shown, representing a non-linear decaying time change.

Supplementary Information 8: There has been recent interest in using capacitive synapses in various devices, which facilitate different materials and memory structures. Table S2 presents a comparative analysis of previous capacitive memory devices along with their unique features. Although some studies have reported on the optical performance of these synapses, it is crucial to note that such devices are still relatively uncommon, particularly in the development of memcapacitance synapses associated with 2D materials. In addition, Fig. S6 illustrates the enhanced capabilities of this work.

**[12-14]]**

**[11]]**

**[15]]**

**[6-8,10]**

**Fig. S6 Capacitive Memory Devices along with their unique features.**

**Table S2|** Table of comparison with previously reported capacitive memory devices, their working mechanism, and applications.

| **Structure/ Materials** | **Working Mechanism** | **Capacitive Memory Modulation** | **Operating Conditions** | **Endurance/**  **Retention** | **Neuron Model implemented** | **Memory Volatility Tunability** | **Application** | **REF** |
| --- | --- | --- | --- | --- | --- | --- | --- | --- |
| **Metal-Ferroelectric-Metal** TiN/Al/Al-doped $\mathrm{HfO}_{2}$ /TiN | Memcapacitor | Electrical | ±2V ̶ ±4V | 10^9^ cycles /  NA | No | No | Accuracy of 78.28% in ANN | ^16^ |
| **Metal-Ferroelectric-Metal** TiN/ (HZO)/TiN | Memcapacitor | Electrical | ±3V for 1ms | 1000 pulses/  10 years at 85C. | No | No | Analog-Shift-and-Add circuits with 26.9% lower energy consumption | ^17^ |
| **Metal-Ferroelectric-Metal** Au/ (HZO)/Au | Memcapacitor | Electrical | ±6V at 50kHz | NA/NA | No | No | Boltzmann machine to reconstruct incomplete images and handwritten digits recognition with 93.4% accuracy. | ^18^ |
| **Conventional N-type FET**  Gate-to-Source/Drain Capacitance | Memcapacitor | Electrical | ±3.5V at 1MHz | NA/NA | No | No | Array architecture of capacitive synapses | ^19^ |
| **Bilayer oxide-based memcapacitor** Pt/ $\mathrm{ZrO}_{X}$/ $\mathrm{WO}_{X}$/ Ta | Memcapacitor | Electrical | ±0.1V- ±3.4V | 100 switching cycles/  20 sec | No | No | Image recognition accuracy of 96.93% with MLP and 99.01% with LeNet-5 | ^20^ |
| **Metal–Semiconductor –Metal** Au/ $\mathrm{La}_{1.875}\mathrm{Sr}_{0.125}\mathrm{NiO}_{4}$/ Au | Memcapacitor | Electrical/Optical | Optically: 365 nm,  Intensity: 20-220 mWcm^-2^  Electrical erasing: pulse  40 V for 10 sec | Optically: 1000 cycles/  1000 sec | No | No | Interest-modulated visual memory | ^21^ |
| **Silicon-on-Insulator** /$\mathrm{Hf}_{0.5}\mathrm{Zr}_{0.5}O_{2}$ | Charge Shielding | Electrical | ±0.6V- ±4V | 10^5^ cycles/  10^2^ sec | No | No | Parallel Multiply–Accumulate Operations with 29,600 tera-operations per second per watt | ^22^ |
| **MOS Memory** P−Si/$\mathrm{SiO}_{2}$/SiN/$\mathrm{SiO}_{2}$/ n+ poly-Si | Charge trapping | Electrical | ±14V for 100μ sec | NA/NA | No | No | Image Recognition Accuracy of 98% | ^23^ |
| **MOS Memory** P− Si/$\mathrm{SiO}_{2}$/ $\mathrm{Si}_{3}N_{4}$/$\mathrm{Al}_{2}O_{3}$ /TiN (TANOS). | Charge trapping | Electrical | ±9V-±18V for 100μ sec | NA/NA | No | No | vector-matrix multiplication (VMM) function and recognition accuracy 99.48%. | ^24^ |
| **MOS Memory** Si/$\mathrm{Al}_{2}O_{3}$/$\mathrm{MoS}_{2}$ /$\mathrm{Al}_{2}O_{3}$/Al | Charge trapping | Electrical/  Optical | Electrical: +6V/−6V, 1 μs  Optical wavelength:  600, 550, 500, 450, and 400 nm,  Intensity: 50 mW cm^-2^ | Electrically:  10^6^ cycles/  10 years at 100°C  Optically programmed/  Electrically erased: 1000 cycles | No | No | Image Recognition Accuracy of 91% | ^25^ |
| **MOS Memory** Si/$\mathrm{Al}_{2}O_{3}$/$\mathrm{HfSe}_{2}$/$\mathrm{Al}_{2}O_{3}$/ITO | Charge trapping and memcapacitor | Electrical/  Optical | Electrical: ±4V- ±10V, 100kHz  Optical wavelength:  465, 532, 635, and 785 nm,  Intensity: 0.8-1051 mW cm^-2^ of 465nm | Electrically:  10^4^ cycles/  10 years at 74°C  Optically programmed/  Electrically erased: 500 cycles | Yes | Yes | Adaptive LIF neuron and Exoplanet detection with 89% accuracy | This work |

**NA**: not mentioned in the reported study.

**References**

1. Crowell, S. Memory: From Mind to Molecules. *Issues in Applied Linguistics* **12**, (2001).

2. Nayfeh, A. & El-Atab, N. Basics of memory devices. in *Nanomaterials-Based Charge Trapping Memory Devices* 1–22 (Elsevier, 2020). doi:10.1016/b978-0-12-822342-0.00001-8.

3. Corrigendum: 1D versus 3D quantum confinement in 1-5 nm ZnO nanoparticle agglomerations for application in charge-trapping memory devices (Nanotechnology (2016) 27 (275205) DOI: 10.1088/0957-4484/27/27/275205). *Nanotechnology* vol. 27 Preprint at https://doi.org/10.1088/0957-4484/27/41/419501 (2016).

4. El-Atab, N. *et al.* Cubic-phase zirconia nano-island growth using atomic layer deposition and application in low-power charge-trapping nonvolatile-memory devices. *Nanotechnology* **28**, (2017).

5. El-Atab, N., Cimen, F., Alkis, S., Okyay, A. K. & Nayfeh, A. Enhanced memory effect with embedded graphene nanoplatelets in ZnO charge trapping layer. *Appl Phys Lett* **105**, (2014).

6. Acharya, J., Wilt, J., Liu, B. & Wu, J. Probing the Dielectric Properties of Ultrathin Al/Al2O3/Al Trilayers Fabricated Using in Situ Sputtering and Atomic Layer Deposition. *ACS Appl Mater Interfaces* **10**, 3112–3120 (2018).

7. Kies, * R, Papadas, C., Pananakakis, G. & Ghibaudo, G. *507 TEMPERATÜRE DEPENDENCE OF FOWLER-NORDHEIM EMISSION TUNNELING CURRENT IN MOS STRUCTURES*.

8. Toumi, S., Ouennoughi, Z. & Murakami, K. Effect of temperature on the Fowler-Nordheim barrier height, flat band potentials and electron/hole effective masses in the MOS capacitors. *Physica B Condens Matter* **585**, (2020).

9. Yue, R. *et al.* HfSe2 thin films: 2D transition metal dichalcogenides grown by molecular beam epitaxy. *ACS Nano* **9**, 474–480 (2015).

10. Huang, M. L. *et al.* Energy-band parameters of atomic layer deposited Al2O 3 and HfO2 on InxGa1-xAs. *Appl Phys Lett* **94**, (2009).

11. Nohira, H. *et al.* *Characterization of ALCVD-Al 2 O 3 and ZrO 2 Layer Using X-Ray Photoelectron Spectroscopy*. www.elsevier.com/locate/jnoncrysol.

12. Magleby, K. L. The effect of repetitive stimulation on facilitation of transmitter release at the frog neuromuscular junction. *J Physiol* **234**, 327–52 (1973).

13. Chang, T., Jo, S. H. & Lu, W. Short-term memory to long-term memory transition in a nanoscale memristor. *ACS Nano* **5**, 7669–7676 (2011).

14. Wang, S. *et al.* Bio-Voltage Memristors: From Physical Mechanisms to Neuromorphic Interfaces. *Advanced Electronic Materials* vol. 9 Preprint at https://doi.org/10.1002/aelm.202200972 (2023).

15. Atluri, P. P. & Regehr, W. G. *Determinants of the Time Course of Facilitation at the Granule Cell to Purkinje Cell Synapse*. (1996).

16. Zheng, Q. *et al.* Artificial Neural Network Based on Doped HfO2 Ferroelectric Capacitors with Multilevel Characteristics. *IEEE Electron Device Letters* **40**, 1309–1312 (2019).

17. Hur, J. *et al.* Nonvolatile Capacitive Crossbar Array for In‐Memory Computing. *Advanced Intelligent Systems* **4**, (2022).

18. Zhu, Y. *et al.* HfZrOx-based capacitive synapses with highly linear and symmetric multilevel characteristics for neuromorphic computing. *Appl Phys Lett* **120**, (2022).

19. Kim, T. H. *et al.* Tunable Non-volatile Gate-to-Source/Drain Capacitance of FeFET for Capacitive Synapse. *IEEE Electron Device Letters* (2023) doi:10.1109/LED.2023.3311344.

20. Lin, P. E. *et al.* Bilayered Oxide Heterostructure-Mediated Capacitance-Based Neuroplasticity Modulation for Neuromorphic Classification. *Adv Funct Mater* (2023) doi:10.1002/adfm.202307961.

21. Zhao, L. *et al.* An Artificial Optoelectronic Synapse Based on a Photoelectric Memcapacitor. *Adv Electron Mater* **6**, (2020).

22. Demasius, K. U., Kirschen, A. & Parkin, S. Energy-efficient memcapacitor devices for neuromorphic computing. *Nat Electron* **4**, 748–756 (2021).

23. Kwon, D. & Chung, I. Y. Capacitive Neural Network Using Charge-Stored Memory Cells for Pattern Recognition Applications. *IEEE Electron Device Letters* **41**, 493–496 (2020).

24. Hwang, S. *et al.* Capacitor-Based Synaptic Devices for Hardware Spiking Neural Networks. *IEEE Electron Device Letters* **43**, 549–552 (2022).

25. Kumar, D. *et al.* Artificial visual perception neural system using a solution-processable MoS2-based in-memory light sensor. *Light Sci Appl* **12**, 109 (2023).
